# Direct-Acting Antivirals Reduce the De Novo Development of Esophageal Varices in Patients with Hepatitis C Virus Related Liver Cirrhosis

**DOI:** 10.3390/v15010252

**Published:** 2023-01-16

**Authors:** Yung-Yu Hsieh, Wei-Ming Chen, Kao-Chi Chang, Te-Sheng Chang, Chao-Hung Hung, Yao-Hsu Yang, Shui-Yi Tung, Kuo-Liang Wei, Chen-Heng Shen, Cheng-Shyong Wu, Yuan-Jie Ding, Jing-Hong Hu, Yu-Ting Huang, Meng-Hung Lin, Chung-Kuang Lu, Yi-Hsiung Lin, Ming-Shyan Lin

**Affiliations:** 1 Division of Gastroenterology and Hepatology, Department of Internal Medicine, Chang Gung Memorial Hospital, Chiayi 613016, Taiwan; 2 College of Medicine, Chang Gung University, Taoyuan 333323, Taiwan; 3 Division of Hepatogastroenterology, Department of Internal Medicine, Kaohsiung Chang Gung Memorial Hospital and Chang Gung University College of Medicine, Kaohsiung 833253, Taiwan; 4 Health Information and Epidemiology Laboratory, Chang Gung Memorial Hospital, Chiayi 613016, Taiwan; 5 Department of Traditional Chinese Medicine, Chang Gung Memorial Hospital, Chiayi 613016, Taiwan; 6 School of Traditional Chinese Medicine, College of Medicine, Chang Gung University, Taoyuan 33302, Taiwan; 7 Division of Gastroenterology and Hepatology, Department of Internal Medicine, Chang Gung Memorial Hospital, Yunlin 638502, Taiwan; 8 Graduate Institute of Education, Taiwan Shoufu University, Tainan 72153, Taiwan; 9 Department of Cardiology, Chang Gung Memorial Hospital, Chiayi 613016, Taiwan

**Keywords:** liver cirrhosis, esophageal varices, portal hypertension, direct-acting antiviral, sustained virologic response

## Abstract

The real-world benefits of direct-acting antiviral (DAA)-induced sustained virologic response (SVR) on the de novo occurrence and progression of esophageal varices (EV) remain unclear in patients with hepatitis C virus (HCV)-related liver cirrhosis (LC). This is a retrospective cohort study evaluating all patients with Child-Pugh class A HCV-related LC during 2013 to 2020 in the Chang Gung Medical System. A total of 215 patients fit the inclusion criteria and were enrolled. Of them, 132 (61.4%) patients achieved DAA induced-SVR and 83 (38.6%) did not receive anti-viral treatment. During a median follow-up of 18.4 (interquartile range, 10.1–30.9) months, the 2-year incidence of de novo EV occurrence was 8 (7.0%) in the SVR group and 7 (12.7%) in the treatment-naïve group. Compared to the treatment-naïve group, the SVR group was associated with a significantly lower incidence of EV occurrence (adjusted hazard ratio [aHR]: 0.47, *p* = 0.030) and a significantly lower incidence of EV progression (aHR: 0.55, *p* = 0.033). The risk of EV progression was strongly correlated with the presence of baseline EV (*p* < 0.001). To the best of our knowledge, this is the first study to demonstrate that DAA-induced SVR is associated with decreased risk of de novo EV occurrence and progression in the real world.

## 1. Introduction

The natural history of patients with hepatitis C virus (HCV)-related compensated liver cirrhosis is now well characterized [1]. About 24% to 80% of patients with liver cirrhosis present with varices [2]. The development of hemorrhagic esophageal varices (EV)—a consequence of portal hypertension—is a major cause of cirrhosis-related morbidity and mortality [3]. Despite major improvements in management, mortality remains as high as 15% after the first episode of EV bleeding [4]. In a prospective study, the rate of incidence of EV was found to be 5% by year 1 and 28% by year 3, and the rate of EV progression was 12% at year 1 and 31% at year 3 for patients with cirrhosis and small EV [5]. The patients with small varices upon enrolment had a higher two-year risk of bleeding from EV than those without varices (12% vs. 2%) [5].

Predicting the development, bleeding, and mortality of EV in patients with cirrhosis is a key concern in clinical practice. It is well established that sustained virologic response (SVR) after interferon (IFN)-based anti-HCV treatment can prevent de novo EV development in patients with HCV-induced cirrhosis [5]. Since 2017, IFN-free direct-acting antiviral (DAA) drugs with a rate of SVR superior to that obtained from IFN-based treatment have been reimbursed by National Health Insurance (NHI) in Taiwan for treating HCV [6]. DAAs showed high efficacy against HCV infection. Pan-genotypic regimens, such as glecaprevir/pibrentasvir (GLE/PIB) and sofosbuvir/velpatasvir (SOF/VEL), showed particular promise during previous clinical studies focused on Taiwanese patients [7,8]. DAA-induced SVR was associated with improvements in liver stiffness and portal hypertension, which could be translate clinically into reductions in hepatic decompensation [9,10,11]. However, the effect of DAA-induced SVR on de novo EV development and progression was not clear. Although a previous study demonstrated that DAAs reduced the risk of bleeding from EV [12], this study included patients with alcoholism, hepatitis B-related cirrhosis, and cryptogenic cirrhosis. To our knowledge, no study has yet assessed the incidence of de novo occurrence and progression of EV following DAA-induced SVR.

## 2. Materials and Methods

### 2.1. Case Enrollment and Data Organization

The present retrospective cohort study, from January 2013 to December 2020, consists of a subgroup analysis based on electronic medical records obtained from the Chang Gung Research Database (CGRD), which is a de-identified and anonymous database [13,14]. The information comes from the various hospitals of the Chang Gung Medical System, the largest hospital system in Taiwan. The patient selection criteria are as follows: (i) presence of anti-HCV antibody and detectable HCV RNA; (ii) cirrhosis diagnosed for the first time during routine surveillance on transient elastography (TE) [15], acoustic radiation force impulse (ARFI) [16], ultrasound [17,18], and histology; and (iii) Child-Pugh class A. The exclusion criteria are as follows: (i) concurrent hepatitis B or human immunodeficiency virus infection; (ii) alcoholism; (iii) previous episodes of decompensation, defined as ascites, spontaneous bacterial peritonitis, encephalopathy, gastroesophageal varices, and hepatorenal syndrome; (iv) previous or active hepatocellular carcinoma (HCC), or cholangiocarcinoma (CCC); (v) previous local treatment for EV, such as EV ligation or endoscopic injection sclerotherapy (EIS); (vi) incomplete model for end-stage liver disease (MELD) score; and (vii) patients without SVR in the DAA group.

**Figure 1:**
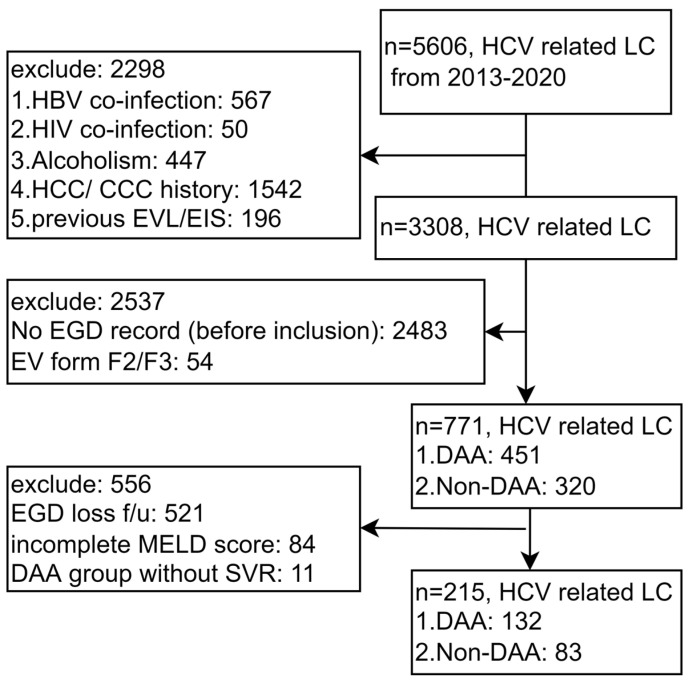
Flowchart of the study. Abbreviations: EGD, esophago-gastro-duodenoscopy; HCV, hepatitis C virus; LC, liver cirrhosis; DAA, direct-acting antivirals; HBV, hepatitis B virus; HIV, human immunodeficiency virus; EVL, esophageal varices ligation, EIS, endoscopic injection sclerotherapy; HCC, hepatocellular carcinoma; CCC, cholangiocarcinoma; f/u follow up; MELD score, Model for End-Stage Liver Disease score; SVR, sustained virologic response.

A total of 5606 patients with HCV-related liver cirrhosis were enrolled. We excluded patients with confounding factors, including 567 patients with HBV co-infection, 50 patients with HIV co-infection, 447 patients with alcoholism, 1542 patients with a history of HCC/CCC, and 196 patients who underwent previous EVL/EIS treatment. We also excluded 2483 patients without a record of esophago-gastro-duodenoscopy (EGD) prior to inclusion, 54 patients with enlarged EV, 521 patients without follow-up data to evaluate de novo occurrence and progression of EV, 84 patients without a complete MELD score, and 11 patients without SVR in the DAA group (Figure 1).

### 2.2. EV and Endoscopy Criteria

The present study only considered patients who had undergone endoscopy prior to the time of enrollment (within 6 months) and were found to be EV-free or display a F1 degree of severity (grade 1 EV). Patients with EV severity of F2 or worse were excluded. Endoscopic procedures for EV assessment were performed by skilled endoscopists, and EV size was determined via medium insufflation and classified according to the North Italian Endoscopic Club score [19].

### 2.3. EV Occurrence/Progression

Retrospective endoscopic observation for EV was logged according to the following definitions. EV occurrence was defined as the development of EV in previously EV-free (F0) patients. EV progression was defined as the occurrence of F1~F3 EV in previously F0 patients, the occurrence of F2~F3 EV in previously F1 patients, or the onset of portal hypertension (PHT)-related bleeding episodes.

### 2.4. SVR in the DAA Group

Designation of patients in the DAA group to receive anti-HCV treatment was determined at the discretion of the treating physician on the basis of the labels approved by the Taiwan Food and Drug Administration and in compliance with the standard of care recommended by international guidelines for HCV infection [20]. SVR is defined as undetectable HCV RNA 12 weeks after the completion of DAA therapy [20].

### 2.5. Statistical Analysis

We considered inverse probability of treatment weighting (IPTW) with propensity score to estimate treatment efficacy in this study [21]. The IPTW-ATE (Average Treatment Effect) weighting method was used to account for variables such as age, sex, diabetes, dyslipidemia, arterial hypertension, obesity, non-carvedilol beta blocker usage, HCV genotype 1b, thrombocytopenia, MELD score, and/or initial EV form. The standardized mean difference (SMD) index was considered to evaluate whether these variables were sufficiently accounted for. When the absolute value of SMD is less than 0.1, it is accepted that there is no difference between the distributions of treatment modalities.

The incidence and progression of varices were visualized as Kaplan-Meier plots. Baseline demographic, clinical, and biochemical parameters, as well as ultrasonographic and endoscopic findings, were analyzed as possible predictors of study endpoints. Multivariate analyses were conducted using a Cox proportional hazards regression model. Results are presented as the adjusted hazard ratio (aHR) with the accompanying 95% confidence interval (CI) after adjustment for potential confounding factors. *p*-values are two-sided with *p* < 0.05 taken as statistically significant. All analyses were performed using SAS version 9.4 (SAS Inc., Cary, NC, USA).

## 3. Results

### 3.1. Baseline Case Characteristics

As demonstrated in Table 1, patients treated with DAAs (n = 132, 61.4%) and those treated without DAAs (n = 83, 38.6%) were of similar mean age (63.2 vs. 63.5 years, SMD = −0.057) and sex (49.3% vs. 46.5% male, SMD = −0.026) after IPTW. Comorbidities such as DM (43.9% vs. 43.3%, SMD = 0.013), dyslipidemia (32.2% vs. 29.1%, SMD = 0.067), arterial hypertension (58.7% vs. 57.0%, SMD = 0.035), obesity (6.0% vs. 5.7%, SMD = 0.009), history of beta blocker usage (6.1% vs. 6.9%, SMD = 0.033), HCV genotype 1b distribution (47.7% vs. 49.2%, SMD = −0.030), thrombocytopenia (49.3% vs. 49.3%, SMD = −0.016), MELD score (7.3 vs. 7.0, SMD < 0.001), and baseline EV F0 distribution (78.0% vs. 78.8%, SMD = −0.002) were also similarly distributed after IPTW. The median follow-up period was 18.4 months (interquartile range 10.1–30.9 months).

### 3.2. Association between DAA and EV Occurrence/Progression

**Figure 2:**
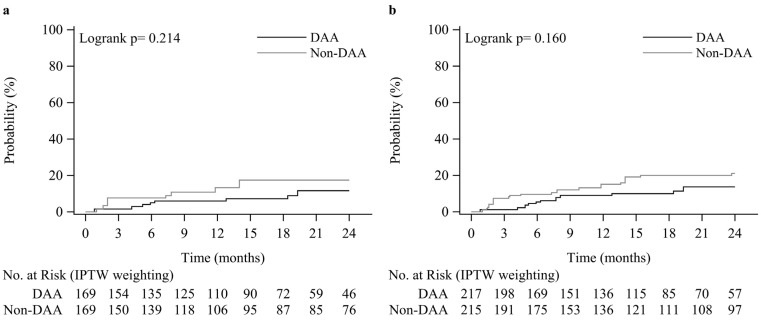
Kaplan-Meier plots of the EV occurrence rate (**a**) and EV progression rate (**b**).

As shown in Figure 2a, the DAA group showed a lower rate of EV occurrence compared to that of the non-DAA group, with incidences of 6.0% vs. 13.3% at year 1, and 11.6% vs. 17.5% at year 2. Multivariable Cox proportional hazards model analysis after IPTW revealed that DAA usage (aHR 0.47, 95% CI 0.24–0.93, *p* = 0.030), MELD score (aHR 1.08, 95% CI 1.02–1.14 for 1 unit increase, *p* = 0.006), and female gender (aHR 22.5, 95% CI 5.58–91.1, *p* < 0.001) were significantly associated with the occurrence of EV (Table 2). Neither HCV genotype 1b nor thrombocytopenia was associated with the occurrence of EV.

As shown in Figure 2b, the DAA group showed a lower rate of EV progression compared to that of the non-DAA group, with incidences of 9.0% vs. 13.7% at year 1, and 15.1 % vs. 21.1% at year 2. Multivariable Cox proportional hazards model analysis after IPTW revealed that DAA usage (aHR 0.55, 95% CI 0.32–0.95, *p* = 0.033), baseline EV of grade F1 (vs. F0) (aHR 3.12, 95% CI 1.72–5.67, *p* < 0.001), female gender (aHR 6.53, 95% CI 2.98–14.3, *p* < 0.001), arterial hypertension (aHR 2.48, 95% CI 1.31–4.71, *p* = 0.006), and diabetes (aHR 2.23, 95% CI 1.21–4.13, *p* = 0.010) were significantly associated with EV progression (Table 3). Neither MELD score, HCV genotype 1b, nor thrombocytopenia was associated with the progression of EV.

### 3.3. Progression of EV as a Function of DAA and Baseline EV Form

**Figure 3:**
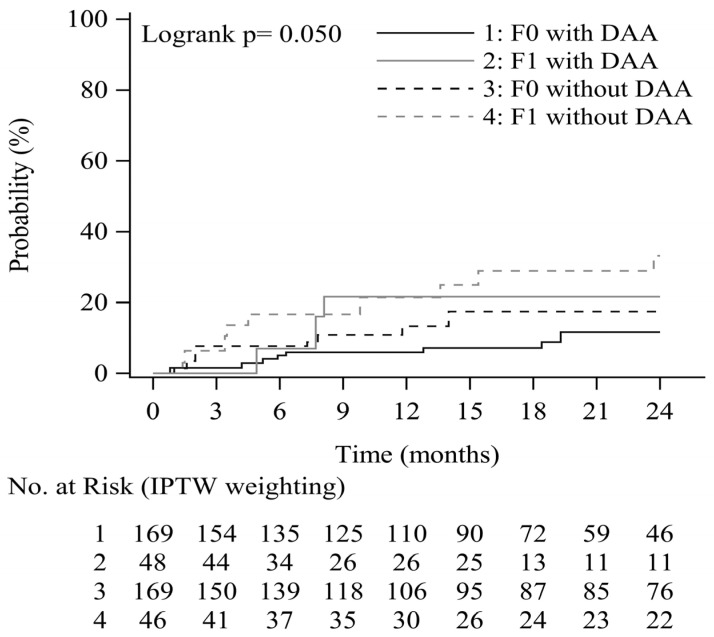
EV progression rate by EV form and DAA treatment.

The cumulative probabilities of EV progression according to endoscopic findings at inclusion and the achievement of viral suppression (DAA induced SVR) are shown in Figure 3. The cumulative progression of EV at 0.5, 1, and 2 years was 5.0%, 6.0%, and 11.6%, respectively, among patients with no EV at inclusion and to whom DAA was administered, and 7.7%, 13.3%, and 17.5%, respectively, among patients with no EV at inclusion and to whom DAA was not administered. Similarly, cumulative progression at these time points was 7.0%, 21.6%, and 21.6%, respectively, among patients with F1 EV at inclusion and to whom DAA was administered, and 16.7%, 21.4%, and 33.2%, respectively, among patients with F1 EV at inclusion and to whom DAA was not administered. DAA-induced SVR and the absence of EV at inclusion were associated with a potential reduced risk of EV progression in patients with HCV-related liver cirrhosis (*p* = 0.050).

## 4. Discussion

This real-world multicenter retrospective study is based on a cohort of patients with HCV-related early cirrhosis who had received DAAs treatment or not. According to methods for comparative effectiveness, we used our observational data to emulate a hypothetical randomized trial by comparing DAA-exposed versus DAA-unexposed patients [22]. Its results show that patients treated with DAAs had significantly lower de novo EV occurrence and progression than those who did not receive DAAs. To the best of our knowledge, the benefit of DAAs on EV was demonstrated for the first time in our IPTW-ATE with propensity score analyses adjusted for demographic, liver function, and comorbidity-related characteristics.

DAAs are accepted as the standard of HCV care by many clinicians [23]. Therefore, it is impracticable to compare the patients who did versus those did not receive DAAs in a randomized controlled trial (RCT) design. As a result, the DAA and non-DAA groups may be compared using the IPTW-ATE method to correct for potential confounding.

The natural development and progression of EV are affected by many clinical factors such as the initial variceal status, EV ligation history, alcoholism, HBV or HIV coinfection, and hepatocellular carcinoma. We herein excluded these confounding factors to estimate causality due to treatment in this observational study. Other factors that may be involved in the development of EV, such as HCV genotype 1b [5], MELD score [5], and thrombocytopenia [24], were also pre-balanced.

In patients with pre-treatment cirrhosis, HCV eradication with SVR may stop progression or even induce regression of the fibrosis spontaneously, leading to improved portal hypertension and lower risk of variceal bleeding [25]. Savino et al. reported that HCV genotype 1b is an independent predictor of EV occurrence [5]. However, the higher occurrence of EV may be due to the lower IFN-induced SVR rate in patients with HCV genotype 1b [26], in which nonstructural 5A gene quasispecies mutations matters. In this study, HCV genotype is not a predictor owing to pangenotypic DAA with high potency.

The baseline MELD score was also an independent predictor of EV, which was noted by Bruno et al. [5] Similarly, in our study, the MELD score was significantly correlated with de novo EV occurrence (aHR 1.08; 95% CI 1.02–1.14 for 1 unit increase, *p* = 0.006), but not with EV progression (*p* = 0.299).

Sanyal et al. reported that the risk of having varices increases with decreasing platelet counts [24]. In our study, thrombocytopenia (platelet counts < 150,000/μL) has no significant correlation with de novo EV occurrence, nor with EV progression. This result is comparable with Qamar et al., who reported that “Platelet count is not a predictor of the presence or development of gastroesophageal varices in cirrhosis” [27].

DAA-induced SVR reduced the risk of EV bleeding in HCV-related liver cirrhosis patients in one clinical study [12]. Our study builds upon this work by considering key initial EV status data. We focus on de novo development of EV in only ethnic Asian patients to exclude confounding factors. In the previous study, data were obtained from the national Veterans Affairs (VA) healthcare system, which is composed of predominantly male (98.1%) patients. In the VA healthcare system, 50.3% of subjects consumed alcohol, and the ethnic makeup was 98% white, black, and Hispanic. Despite differences between the VA healthcare and our database, both studies infer that DAA reduces the risk of EV occurrence, progression, and bleeding. This has led to the hypothesis that DAA halts progression of HCV-related liver cirrhosis.

Besides SVR, metabolic syndrome also affects the natural history of PHT in cirrhosis [28]. In the previous IFN-based treatment for HCV, the patients with insulin resistance (type 2 DM) may be at higher risk for worse outcomes including a reduced SVR rate and early progression to fibrosis and cirrhosis [29,30]. In this study, DM (aHR 2.23, *p* = 0.010) and arterial hypertension (aHR 2.48, *p* = 0.006) have significant correlation with EV progression, but not EV occurrence. Morbidities of other metabolic syndromes such as obesity and dyslipidemia had no significant correlation with EV occurrence or progression.

Female gender correlated significantly with de novo EV occurrence and progression (*p* < 0.001) in this study. Khan et al. reported that women display a significantly different EV bleeding rate after particular treatment [31]. The possible mechanism may be due to hormones and pregnancy. Estrogen-increased venous capacitance and progesterone-weakened blood vessel walls were reported [32]. Pregnancy doubles blood volume in order to supply the baby. The increase in blood volume applies stress to the vessels and may cause varices [33].

According to the 2016 practice guidance about portal hypertensive bleeding in cirrhosis by the American Association for the Study of Liver Diseases (AASLD) [34], there is no evidence to support recommending non-selective beta blockers (NSBBs) in patients without varices. However, the primary prophylaxis of variceal hemorrhage is indicated for patients with small varices with red wale signs or decompensation. In clinical practice, early NSBBs to prevent EV development seems common particularly when hepatic venous pressure gradient (HVPG) information is not available. Despite that, this kind of practice may only be beneficial while administering carvedilol [35]. Other NSBBs, except for carvedilol, show a significantly higher incidence of adverse events. Nevertheless, these drugs offer no significance against EV development, upper gastrointestinal bleeding, or death [36]. In our study, non-carvedilol beta blockers showed potential higher EV progression (aHR 2.18, *p* = 0.051), which may be because patients with high-risk varices (e.g., red wale markings and enlarged EV) tend to also receive NSBBs. Conversely, NSBBs can not only be used to prevent variceal bleeding, but also to treat arterial hypertension, angina, tremor, and migraine; therefore, they are not considered specific enough. Hence, the use of NSBBs may be a potential confounder.

There are some limitations of this study. First, this is a study from Taiwan, where genotypes 4 and 5 are rare. Second, clinical data were not complete in this retrospective study and a majority of patients were therefore excluded. According to the flow chart (Figure 1), most of the patients were excluded because they did not undergo initial EGD or their EV condition was already very serious, which accounted for 2537 patients. In hope of proving that the included group was representative to the whole cohort, the Supplement Table S1 compared the differences between the “excluded” and “included” groups by the basic variables listed in “Table 1”. The numbers were divided into the included (n = 215) and excluded groups (n = 3093) from the total of 3308 in the flow chart (Figure 1). The result revealed that the only difference between the two groups was age. Other factors, such as comorbidity factors (DM, dyslipidemia, arterial HTN, obesity), platelets, MELD score, Fib4 score and DAA using were not significantly different (*p* > 0.05). Third, each modality for evaluating liver cirrhosis has particular shortcomings.

## 5. Conclusions

Despite early cirrhosis, SVR after DAAs significantly reduced the de novo occurrence and progression of EV during the short-term 24-month period study. In clinical practice, frequent EGD observation may be avoided in SVR patients. This study provides further evidence supporting the real-world benefits of early DAA treatment in HCV-positive patients with cirrhosis of the liver.

## Figures and Tables

**Table 1:** Baseline characteristics of patients.

Variables	Unweighted Sample, No.				SMD	IPTW ^†^ Sample		SMD
	DAA		Non-DAA			DAA	Non-DAA	
	(*n* = 132)		(*n =* 83)			(*n* = 217)	(*n* = 215)	
Sex					0.033			−0.057
Female	69	(52.3%)	42	(50.6%)		50.7%	53.5%	
Male	63	(47.7%)	41	(49.4%)		49.3%	46.5%	
Age (years)								
Mean (±SD)	64.8	(±10.3)	61.0	(±9.7)	0.378	63.2	63.5	−0.026
Comorbidity								
Diabetes	55	(41.7%)	38	(45.8%)	−0.083	43.9%	43.3%	0.013
Dyslipidemia	42	(31.8%)	23	(27.7%)	0.090	32.2%	29.1%	0.067
Arterial hypertension	76	(57.6%)	47	(56.6%)	−0.019	58.7%	57.0%	0.035
Obesity	6	(4.6%)	7	(8.4%)	−0.158	6.0%	5.7%	0.009
Use of non-carvedilol beta blockers	8	(6.1%)	4	(4.8%)	0.055	6.1%	6.9%	−0.033
Baseline EV form					0.509			−0.002
F0	115	(87.1%)	55	(66.3%)		78.0%	78.8%	
F1	17	(12.9%)	28	(33.7%)		22.0%	21.2%	
Genotype					0.127			−0.030
1b	64	(48.5%)	35	(42.2%)		47.7%	49.2%	
Non-1b	68	(51.5%)	48	(57.8%)		52.3%	50.8%	
Platelets (1000/mL)					−0.397			−0.016
<150	54	(40.9%)	48	(57.8%)		49.3%	49.3%	
≥150	78	(59.1%)	35	(42.2%)		50.7%	50.7%	
MELD score								
Mean (±SD)	6.8	(±6.6)	7.1	(±6.1)	0.343	7.3	7.0	0.000
Median (25–75% quartiles)	5	(3–7)	5	(3–9)				
Outcomes								
EV occurrence ^‡^	8	(7.0%)	7	(12.7%)	−0.195	8.0%	14.6%	−0.208
EV progression	11	(8.3%)	16	(19.3%)	−0.321	9.7%	17.7%	−0.233

Abbreviations: IPTW: inverse probability treatment weighting; SD: standard deviation; SMD: standardized mean difference. ^†^ IPTW sample adjusted by sex, age, diabetes, dyslipidemia, arterial hypertension, obesity, non-carvedilol beta blocker using, initially EV form, genotype, platelets, and MELD score. ^‡^ The data were calculated from the initial EV form was F0 cases.

**Table 2:** Multivariable Cox proportional hazards model analysis of factors associated with EV occurrence in HCV patients with liver cirrhosis.

Variables	Unweighted Sample				IPTW Sample			
	Crude HR		Adjusted HR		Crude HR		Adjusted HR	
	HR (95% CI)	*p*-Value	HR (95% CI)	*p*-Value	HR (95% CI)	*p*-Value	HR (95% CI)	*p*-Value
Sex								
Female	5.83 (1.31–25.8)	0.020	7.67 (1.41–41.8)	0.019	11.8 (3.42–40.6)	0.001	22.5 (5.58–91.1)	<0.001
Male	1		1		1		1	
Age (linear) ^†^	1.55 (0.91–2.65)	0.108	1.51 (0.73–3.14)	0.268	1.83 (1.31–2.56)	<0.001	1.35 (0.85–2.15)	0.205
Comorbidity								
Diabetes	2.69 (0.92–7.87)	0.071	1.89 (0.51–7.05)	0.341	2.14 (1.12–4.10)	0.022	1.63 (0.67–3.97)	0.279
Dyslipidemia	0.75 (0.24–2.36)	0.623	0.63 (0.17–2.34)	0.488	0.88 (0.44–1.77)	0.726	0.61 (0.26–1.44)	0.262
Arterial hypertension	1.42 (0.48–4.15)	0.524	0.82 (0.21–3.22)	0.774	2.59 (1.21–5.53)	0.014	2.28 (0.88–5.87)	0.088
Obesity	2.43 (0.56–10.8)	0.242	2.03 (0.33–12.7)	0.448	2.05 (0.73–5.79)	0.176	1.32 (0.38–4.52)	0.661
Use of non-carvedilol beta blockers	2.28 (0.51–10.1)	0.279	1.34 (0.25–7.03)	0.732	1.74 (0.66–4.59)	0.262	0.95 (0.32–2.78)	0.920
Genotype								
1b	0.77 (0.27–2.15)	0.612	0.64 (0.21–1.95)	0.429	0.82 (0.44–1.56)	0.554	0.73 (0.35–1.52)	0.402
Non-1b	1		1		1		1	
Platelets (10^3^/μL)								
<150	2.44 (0.83–7.15)	0.104	1.33 (0.39–4.55)	0.655	1.35 (0.72–2.55)	0.355	1.76 (0.36–1.64)	0.489
≥150	1		1		1		1	
MELD score (linear) ^a^	1.03 (0.97–1.10)	0.322	1.08 (0.99–1.17)	0.053	1.01 (0.97–1.06)	0.569	1.08 (1.02–1.14)	0.006
Treatment								
DAA	0.58 (0.21–1.60)	0.294	0.57 (0.19–1.73)	0.324	0.56 (0.29–1.09)	0.089	0.47 (0.24–0.93)	0.030
Non-DAA	1		1		1		1	

^†^ HRs were calculated by per 10-unit change for age and ^a^ per 1-unit change for MELD scores.

**Table 3:** Multivariable Cox proportional hazards model analysis of factors associated with EV progression in HCV patients with liver cirrhosis.

Variables	Unweighted Sample				IPTW Sample			
	Crude HR		Adjusted HR		Crude HR		Adjusted HR	
	HR (95% CI)	*p*-Value	HR (95% CI)	*p*-Value	HR (95% CI)	*p*-Value	HR (95% CI)	*p*-Value
Sex								
Female	2.85 (1.21–6.74)	0.017	4.35 (1.53–12.4)	0.006	3.83 (2.04–7.22)	<0.001	6.53 (2.98–14.3)	<0.001
Male	1		1		1		1	
Age (linear) ^†^	1.08 (0.74–1.58)	0.676	0.81 (0.49–1.33)	0.403	1.31 (1.01–1.69)	0.044	0.93 (0.67–1.29)	0.645
Comorbidity								
Diabetes	1.50 (0.71–3.20)	0.291	1.61 (0.69–3.74)	0.267	1.68 (1.00–2.80)	0.048	2.23 (1.21–4.13)	0.010
Dyslipidemia	0.42 (0.14–1.20)	0.106	0.59 (0.18–1.93)	0.380	0.56 (0.30–1.08)	0.082	0.54 (0.26–1.12)	0.099
Arterial hypertension	1.17 (0.54–2.53)	0.687	1.62 (0.63–4.21)	0.319	1.80 (1.04–3.13)	0.037	2.48 (1.31–4.71)	0.006
Obesity	1.29 (0.31–5.45)	0.729	1.03 (0.20–5.33)	0.974	1.18 (0.43–3.27)	0.747	0.84 (0.27–2.58)	0.754
Use of non-carvedilol beta blockers	2.94 (1.02–8.52)	0.047	3.30 (0.95–11.4)	0.061	2.74 (1.37–5.48)	0.004	2.18 (1.00–4.76)	0.051
Baseline EV form								
F0	1		1		1		1	
F1	3.02 (1.41–6.45)	0.004	3.62 (1.51–8.71)	0.004	2.07 (1.21–3.53)	0.008	3.12 (1.72–5.67)	<0.001
Genotype								
1b	0.67 (0.31–1.47)	0.318	0.73 (0.31–1.72)	0.475	0.80 (0.48–1.33)	0.384	1.07 (0.60–1.91)	0.810
Non-1b	1		1		1		1	
Platelets (10^3^/μL)								
<150	2.57 (1.12–5.87)	0.025	1.96 (0.80–4.79)	0.141	1.41 (0.84–2.37)	0.189	0.93 (0.53–1.66)	0.813
≥150	1		1		1		1	
MELD score (linear) ^a^	1.02 (0.96–1.07)	0.546	1.05 (0.99–1.12)	0.119	1.00 (0.96–1.04)	0.942	1.02 (0.98–1.07)	0.299
Treatment								
DAA	0.46 (0.21–0.98)	0.045	0.69 (0.30–1.60)	0.391	0.57 (0.34–0.98)	0.040	0.55 (0.32–0.95)	0.033
Non-DAA	1		1		1		1	

^†^ HRs were calculated by per 10-unit change for age and ^a^ per 1-unit change for MELD scores.

## Data Availability

The data that support the findings of this study are available on request from the first author with the permission of the IRB of Chang Gung Memorial Hospital, ChiaYi branch. The data are not publicly available due to privacy or ethical restrictions.

## Supplementary Materials

The following supporting information can be downloaded at: https://www.mdpi.com/article/10.3390/v15010252/s1, Table S1: Comparison of included and excluded participants.
