# Supplementary material for: Direct-Acting Antivirals Reduce the De Novo Development of Esophageal Varices in Patients with Hepatitis C Virus Related Liver Cirrhosis
Source: Viruses. 2023 Jan 16;15(1):252. doi: 10.3390/v15010252 (PMC9860555; doi:10.3390/v15010252)
Supplement: Supplementary file 1 [file viruses-15-00252-s001.zip › viruses-2075984-supplementary.pdf]

Table S1. Comparison of included and excluded participants

Variables	Excluded patiens (*n* = 3093)	Included patiens (n = 215)	P-value
Sex			0.572
Female	1660 (53.7%)	111 (51.6%)	
Male	1433 (46.3%)	104 (48.4%)	
Age (years)			
Mean (±SD)	65.0 (±11.5)	63.3 (±10.2)	0.023
Comorbidity			
Diabetes	1149 (37.2%)	93 (43.3%)	0.080
Dyslipidemia	885 (28.6%)	65 (30.2%)	0.640
Arterial hypertension	1556 (50.3%)	123 (57.2%)	0.057
Obesity	125 (4.0%)	13 (6.1%)	0.157
Non-carvedilol beta blocker using			0.283
No	2969 (96.0%)	203 (94.4%)	
Yes	124 (4.0%)	12 (5.6%)	
Platelets (1000/mL)			0.888
<150	1383 (46.7%)	102 (47.4%)	
≥150	1576 (53.3%)	113 (52.6%)	
Missing	134	0	
FIB4 score			0.352
≤3.25	1678 (57.0%)	114 (53.5%)	
>3.25	1267 (43.0%)	99 (46.5%)	
Missing	148	2	
MELD score			
Mean (±SD)	6.4 (±6.2)	6.9 (±6.4)	0.206
Missing	500	0	
DAA using			0.771
No	1162 (37.6%)	83 (38.6%)	
Yes	1931 (62.4%)	132 (61.4%)	

Abbreviations: SD: standard deviation;
